# Hydrofoil-like legs help stream mayfly larvae to stay on the ground

**DOI:** 10.1007/s00359-023-01620-2

**Published:** 2023-02-26

**Authors:** Petra Ditsche, Florian Hoffmann, Sarah Kaehlert, Antonia Kesel, Stanislav Gorb

**Affiliations:** 1 Department of Functional Morphology and Biomechanics, Zoological Institute, University of Kiel, 24098 Kiel, Germany; 2 Biomimetics-Innovation-Centre, City University of Applied Sciences, 28199 Bremen, Germany; 3 Friday Harbor Laboratories, University of Washington, Friday Harbor, WA 98250 USA; 4 Present Address: ClingTech Bionics UG, 53757 Sankt Augustin, Germany

**Keywords:** *Ecdyonurus* sp., Femur, Lift, Drag, Torrenticol

## Abstract

Adaptations to flow have already been in the focus of early stream research, but till today morphological adaptations of stream insects are hardly understood. While most previous stream research focused on drag, the effects of lift on ground-living stream insects have been often overlooked. Stream mayfly larvae *Ecdyonurus* sp. graze on algae on top of the stones and therefore inhabit current exposed places in streams. They have a dorso-ventrally flattened body shape, which is known to reduce drag. However, this body shape enhances lift too, increasing the danger for the animal of getting detached from the substrate. Using microscopic techniques, 3D-printing, and drag and lift measurements in a wind tunnel, our experiments show that the widened femora of *Ecdyonurus* sp. can generate negative lift, contributing to counterbalance the (positive) lift of the overall body shape. The larvae can actively regulate the amount of lift by adjusting the femur’s tilt or optimizing the distance to the ground. This shows that morphological adaptations of benthic stream insects can be very elaborate and can reach far beyond adaptations of the overall body shape. In the presented case, *Ecdyonurus* sp. takes advantage of the flow to overcome the flow’s challenges.

**Supplementary Information:**

The online version contains supplementary material available at 10.1007/s00359-023-01620-2.

## Introduction

Water velocity and the associated physical forces represent the most important environmental factor affecting organisms in running waters (Allan 1995). Benefiting from the positive effects of water flow on respiration, nutrient, and food supplies (Ruttner 1962), lotic habitats often have more abundant fauna compared to lentic habitats (Schoenborn 1992). However, to benefit from the flow, rheophilic animals must cope with the hydrodynamic impact of flow forces (Allan 1995). Adaptations of benthic (living at the bottom) stream insects to a life in current have been in the focus of interest and the topic of an intensive, often controversial discussion already at the beginning of stream research (Steinmann 1907; Thienemann 1925; Hubault 1927; Wesenberg-Lund 1943; Nielsen 1950a, b; Ruttner 1962). This discussion came to a sudden end when Ambühl (1959) integrated Prandtl’s findings of boundary layers into running water biology. While we know today that strong currents usually do not simply pass over the animal, but directly affect them (Smith and Dartnall 1980; Statzner and Holm 1982, 1989; Weissenberger et al. 1991; Waringer 1993) several basic questions about morphological adaptations to strong currents are still not answered, or experimentally proven (Statzner 2008; Ditsche and Summers 2014). Moreover, most of the former literature focuses only on drag. Aside drag lift is another important force caused by flow (Vogel 1994). While it might be often more challenging for benthic animals to deal with lift than with drag (Statzner and Holm 1982), very little is known about how benthic stream insects are adapted to deal with lift. In general, there are two main strategies of morphological adaptations to deal with currents: (1) benthic stream animals are often equipped with strong attachment devices helping them stay in place (Hora 1936; Hynes 1970; Ditsche and Summers 2014; Kang et al. 2021); (2) drag and lift, which act to dislodge the animal, can be affected by the animal’s body shape, and potentially reduced by the animal (Vogel 1994; Ditsche and Summers 2014).

In this study, we focus on larvae of the heptageniid mayfly family, which are typical inhabitants of fast-flowing streams. These mayfly larvae feed on algae and biofilm growing on the upper side of rocks at the stream bottom, and, therefore, crawl regularly to current-exposed places (Ditsche-Kuru et al. 2010). Heptageniid larvae are usually considered dorso-ventrally flattened (Ward 1992; Merritt and Cummins 1996). A dorso-ventrally flattened body shape is known to reduce the drag acting on the animal. However, it is known that this body shape increases lift as well (Vogel 1994; Statzner 2008). Consequently, the larvae could be expected to run in danger of being lifted from the ground and drifted away downstream.

**Fig. 1:**
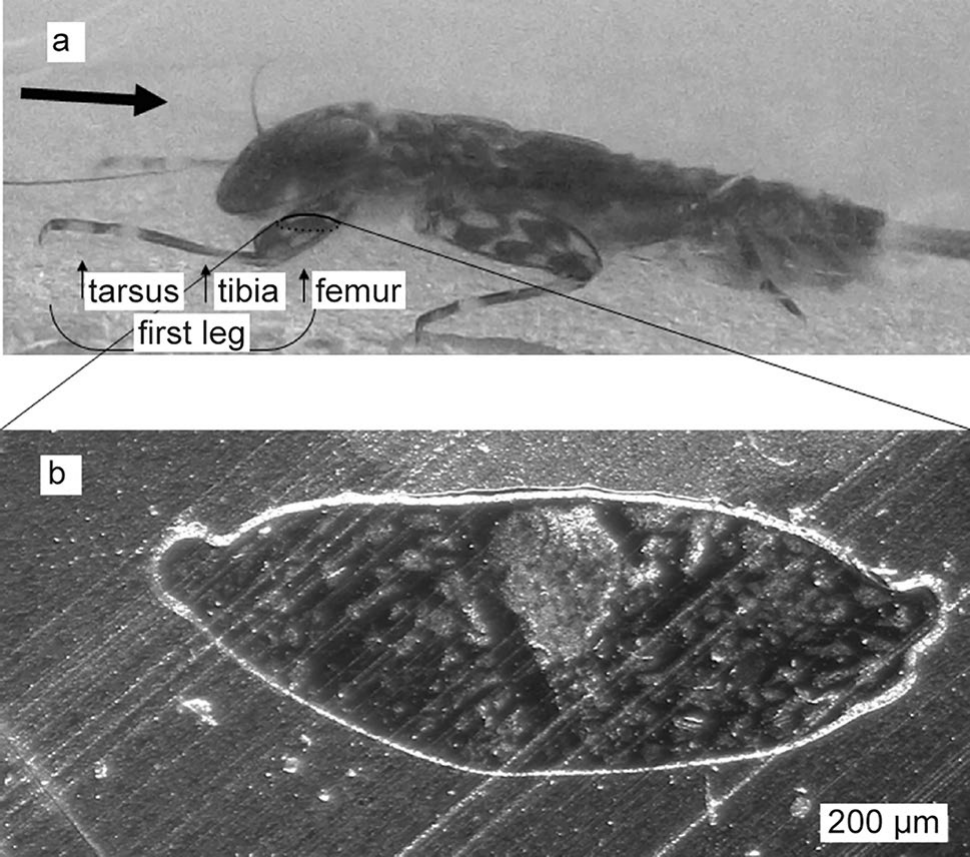
**a** *Ecdyonurus* larva in flow tank. Frame taken from a video. The black arrow shows the direction of flow. The length of the larva is approximately 9 mm. **b** Light microscopy image of a typical cross section of the middle region of the first femur of *Ecdyonurus* sp. embedded in epoxy resin

With increasing flow velocity, heptageniid larvae can be observed to arrange themselves facing the flow and exposing their femora (Fig. 1a), often remaining in a seemingly motionless position for a longer time. In comparison to other parts of the leg, the femora are clearly widened. It is conspicuous that the larvae hold the femora in a way that the area of frontal projection is increased, a position in which drag forces increase. This raises the question if there is an effect on lift, which might be more important for the animal than reducing drag.

For the heptageniid species *Ecdyonurus* sp., it has been suggested that the tilted femora and the lowered head shields can reduce lift (Weissenberger et al. 1991). The same has been suggested for *Penaphlebia* sp., a strikingly similar-shaped leptophlebiid larvae inhabiting South America (Gonser 1990). These assumptions have been supported by measurements of lift on living *Ecdynurus* sp. larvae (Weissenberger et al. 1991). With lift forces ranging from − 1.2 to 1.2 mN (flow velocities 0.5–0.7 m/s), living *Ecdyonurus* sp. larvae experienced not only positive but also negative lift. The same authors introduced the word “spoiler” for the femora of *Ecdyonurus* sp., but the mechanism has never been proven or discussed in detail. The huge variation of lift at comparable flow velocities (Weissenberger et al. 1991) suggests that the larvae can actively affect the lift that they experience.

Some benthic stream fish such as darters or sculpins hold their fins at a certain angle when they hold station on the ground (Carlson and Lauder 2011; Kane and Higham 2012). The same has been observed for marine bamboo sharks (Wilga and Lauder 2001). In this position, a vortex forms behind the fin producing negative lift forces. These negative lift forces push the fish towards the substrate and thereby support its stability (Arnold and Weihs 1978; Wilga and Lauder 2001; Carlson and Lauder 2011). We hypothesize that some benthic stream insects can use their legs in a similar way, like upside-down wings. While 3D-effects are well known to affect the fluid dynamics in swimming or flapping flight, the legs of *Ecdyonurus* sp. remain motionless over longer time periods and are therefore more comparable with steady-state fluid dynamics. For the latter, the use of 2D-wing profiles (airfoils) has a long-standing tradition in engineering to describe the effects of wing shape, angle of attack (AOA), and Reynolds number (Re) for example in the NACA profiles in airplane design. Investigations on flying snakes showed that 2D-modeling can under certain conditions even be applied at slow undulating flight (Holden et al. 2014).

Benthic animals, such as *Ecdyonurus* sp., are in close proximity to the ground, where specific fluid phenomena can be evident (Cui and Zhang 2010). We refer to these specific fluid dynamic phenomena in the following as ground effects, including a velocity gradient forming over a solid body, called the boundary layer. At the substrate surface, the flow velocity is 0 (non-slip condition) and it increases with the distance until it reaches 99% of the free stream velocity (Vogel 1994). Depending on the specific conditions (flow speed, bottom structure, etc.), the thickness and type of the boundary layer vary tremendously. The boundary layer influences directly the flow conditions experienced by the organisms inhabiting the bottom substrate. Depending on their body height in relation to the thickness of the boundary layer, they may be exposed to smaller or larger regions of reduced flow. Second, the proximity to the ground impacts the pressure distribution around approaching wings and vortex development and, therefore, affects lift and drag (Chawla et al. 1990; Ahmed and Sharma 2005; Rozhdestvensky 2006; Abramowski 2007; Cui and Zhang 2010; Bleischwitz et al. 2016). Due to this wing-in-ground effect, airplanes experience typically an increase of lift and a decrease of drag during landing. This is caused by higher pressure under the wing and interference with vortex formation when the plane is approaching the ground. This effect is used by ground effect air crafts (Rozhdestvensky 2006). Third, the distance between a body and the ground can impact the hydrodynamic forces due to the Bernoulli effect. For example, racing cars are generating downwards-directed forces by taking advantage of the Bernoulli effect (Cui and Zhang 2010). Most of the studies on wings in proximity to the ground have been conducted for technical applications and at high Re. Impacts of the ground effects (as defined above) on swimming benthic fish and birds have been also previously described (Webb 1988; Blevins and Lauder 2013; Kim et al. 2014; Quinn et al. 2014). Under free stream conditions, most studies of wing profiles have been performed for higher Re as well (NACA, EPPLER airfoils). Studies on animals in the intermediate Re range (10 < Re < 10,000) are rare, although some measurements have been done for flying snakes and insect-inspired technical wings (Dickinson and Götz 1993; Kesel 2000; Usherwood and Ellington 2002; Holden at al. 2014). The cross-sectional profiles of the latter are not comparable with the profile of the femora of *Ecdyonurus* though.

In this study, we are using microscopical techniques, video observations, 3D-printing, and drag and lift measurements on 2D-wing models in a wind tunnel to investigate, (1) if the femur acts like an upside-down wing (or hydrofoil) and produces a downwards directed force (negative lift), and (2) if this potential effect is affected by the proximity to the ground.

## Materials and methods

### Animal material

We collected *Ecdyonurus* sp. larvae end of April in Schlingenbach stream (Overrath, Germany). Some of the larvae were fixed in 70% ethanol (EtOH), while others were transported alive to a flow tank at the Kiel University. The larvae used in our study were middle to later instars. For identification, the key from Eiseler (2005) was used. The larvae in our study belonged to the *Ecdyonurus venosus* group (*Ecdonurus cf. torrentis*), but reliable identification on species level seems only possible in nymphs, so we refer to them in this study as *Ecdyonurus* sp.

### Cross-sectional femur profile

Although all legs will have an impact on the flow forces, we focused on the first femora, as they are typically most directly exposed to the current (Fig. 1a). Length and chord length of each foreleg femur from 10 different *Ecdyonurus* sp. specimens were measured under a dissecting microscope. For the preparation of cross sections, the right and left first femora of 10 *Ecdyonurus* sp. specimens were embedded in the Epon epoxy resin. For this purpose, we dehydrated the femora of fixed specimens in a series of ethanol (70%, 95%, 100%) and embedded them in Epon following the protocol after Luft (1961). To get semi-hard Epon resin, 15 ml Epon I (124 ml Epon 812, 200 ml Dodecenyl Succinic Anhydride) and 35 ml Epon II (300 ml Epon 812, 267 ml *N*-methyl Nadicanhyrdid) were mixed for 30 min, 0.75 ml Tris-2,4,5-dimethylaminomethylphenol added and mixed for another 30 min. 20 molds were filled with Epon half full and left to polymerise at 60 °C overnight. After infiltration (one part 100% Ethanol dehydrated by molecular sieve: one part Epon for 30 min, one part 100% Ethanol dehydrated by molecular sieve: two parts Epon for 90 min, pure Epon overnight, pure Epon 6–7 h), one leg was put into each mold. Then, the molds were filled up to the top with the pure Epon and left to polymerize at 60 °C for 48 h. The cross sections were prepared from the middle section of the first femur. A typical cross section (Fig. 1b) was chosen for the construction of the femur model.

### Determination of the angle of attack (AOA)

We took video recordings and photos of living *Ecdyonurus* sp. larvae in a flow tank (Fig. 1a). By analyzing video footage and photos, we estimated the AOA of the femora of the living larvae using the program ImageJ (NIH, https://imagej.nih.gov/ij/). As the perspective interferes with the determination of the AOA from 2D-images, we additionally determined the morphologically possible range of the femur’s AOA. Therefore, we placed rehydrated larvae on a polyvinylsiloxane layer (President Light Body, Coltene Whaledent, Hamburg, Germany) at the bottom of a Petri dish and pinned them with insect pins to the rubber-like polymer. To determine the AOA, we measured the possible tilt of the femur in relation to the body axes and the substrate. Three coordinates surrounding the larvae in a triangle were measured to determine the substrate surface plane. Along the longitudinal body axis, we measured three points and one more lateral point on each side of the first body segment to determine the mediolateral axis. The femur position was determined by three points, with point 1 at the femur–tibia joint, and points 2 and 3 outside the femur edge. Points were marked with a drop of red varnish and measured using Mitutoyo Measuring Microscope (MF-A Series, Series 176). 3D coordinates of all points were determined at the minimum and maximum AOAs of the femur while manually changing the position of the femur and securing the position with additional insect pins. The location of the measurement points is illustrated in Supplement 1.

### Femur models

As the larvae often remain motionless in the same position, steady-state fluid dynamics and 2D-modelling are applicable. 2D-wing models were built from the foreleg femur pair of *Ecdyonorus* sp. The contour line of the cross section of the femur’s middle region was digitized using software Rhino 4.0 (McNeel, Seattle, USA), and the cross section was stretched to 250 mm in length. Different sizes of femur models were chosen to investigate not only at a range of the Re number comparable to the natural situation of *Ecdyonurs* sp. (M1), but also to investigate flow forces at higher Re numbers. The latter allowed the comparison with measurements described in the literature, which often have been performed at higher Re, and also can be important for potential technical applications. For all femur models, the cross section was enlarged (Table 1) to obtain Re from 1700 to 24,000 at wind speeds from 2.5 to 6.7 m/s. The femur models were printed using a Contex MX powder-based 3D printer (Contex A/S, Alleroed, Denmark). To smoothen the surface of the raw 3D prints, the surface was filled with body filler, polished with sandpaper and finished with an acrylic spray paint. We also included a flat plate as a reference.

**Table 1:** Geometrical variables and Reynolds numbers (Re) of the first femur of *Ecdyonurus* sp. and the profiles used in this study

	Chord length, *c *(mm)	Profile thickness, *T *(mm)	Span length, *l *(mm)	Fluid	Flow velocity, *U *(m/s)	Reynolds number, Re
First femur of *Ecdyonorus* sp.	~ 1	~ 0.4	~ 2.5	Water	0.1–2	75–1500
Model 1 (M1)	10	4	250	Air	2.5	1700
Model 1 (M1)	10	4	250	Air	6.7	4500
Model 2 (M2)	25	9.6	250	Air	6.7	11,000
Model 3 (M3)	50	18.5	250	Air	6.7	22,000
Flat plate (EP)	68	1	280	Air	6.7	30,000

### Wind tunnel

**Fig. 2:**
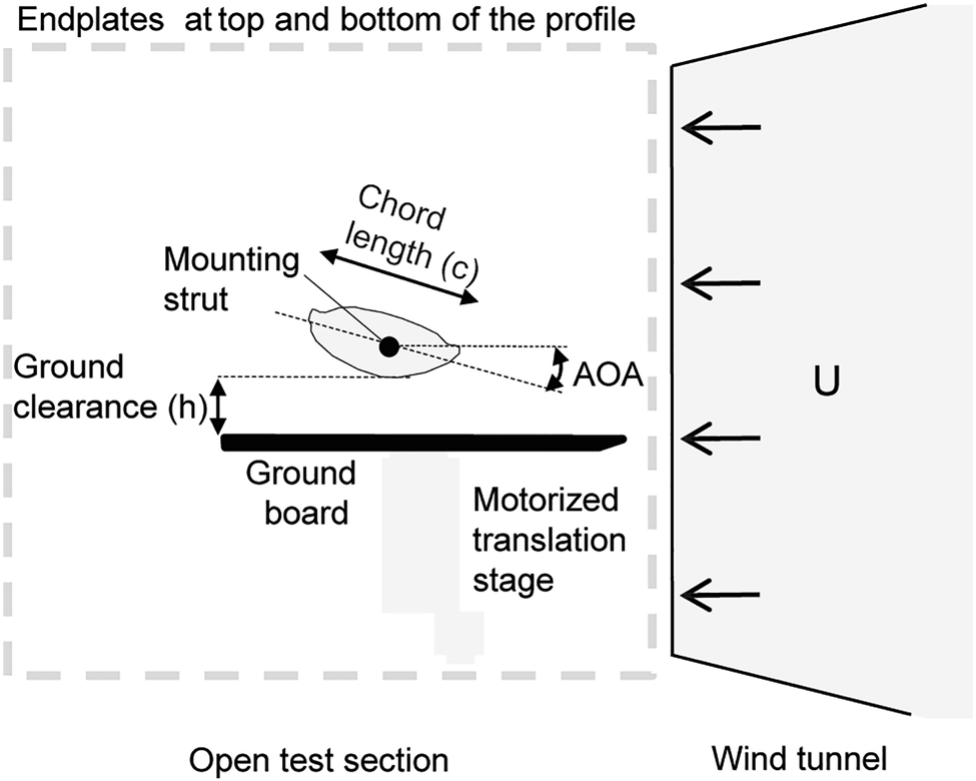
Schematic top view of the experimental setup in the wind tunnel. The artificial femur profile was mounted between two endplates on the two component force balance. The distance between the leading edge of the ground board and the leading edge of the femur profile was 100 mm. The distance to the ground (*h*) varied from 4–60 mm by moving the motorized translation stage mounted to a ground board

Drag and lift forces perform comparably in various fluids, if determined for the same Re (Vogel 1994). For our experiments, we used a custom-made wind tunnel of Eiffel-type (open circuit) with an open test section, nozzle diameter 0.46 m, turbulence 0.3–0.6% (Dickinson and Götz 1993), and adjustable wind speed of 0.5–17.0 m/s (measured by a Pitot tube connected with a digital manometer EMA 200 with a range of ± 200 Pa (Halstrup-Walcher GmbH, Kirchzarten, Germany). The femur models were connected vertically to the force balance (Fig. 2). To limit spanwise flow and induced drag, an endplate on each side of the profile (distance < 0.5 mm) was placed.

The Reynolds number is important to compare the flow conditions. It can be calculated from the equation:

1 $${{\rm{Re}}=\frac{U l}{\nu }},$$

where *l* is a characteristic length (here chord length), *U* the fluid velocity and *ν* the kinematic viscosity (water at 10 °C: 1.3063 × 10^−6^ m^2^ s^−1^, dry air at 20 °C, 1.461 × 10^− 5^ m^2^ s^−1^). All Re for the performed experiments (1700–24,000) were below the critical Re* =* 32,000, where the flow would change from laminar to turbulent. The femora of *Ecdyonorus* larvae have no sharp leading-edge structures, which would evoke a turbulent flow at lower Re, so that laminar flow and the presence of a boundary layer can be expected. *Ecdyonurus* larvae tolerate flow speeds up to 2 m/s (Butz 1975). Re for flow conditions in their natural habitat, which can be calculated from the chord length and the flow speeds described for the natural habitat, range from 75 to 1500. In our experiment, 1700 were considered comparable with the biological case. Pretests revealed that measurements at Re < 1700 reached the limits of the experimental setup due to given limitations in wind tunnel speed, the precision of balance, and model. The higher Reynold numbers in our experiments (> 4500) have no relevance for the biological system, but is of interest for comparison of the aero- or hydrodynamic characteristics of the femur profile with other profiles, such as the widely applied NACA profiles, and for the potential transfer to biomimetic technical solutions.

The blocking of the femur model, mounted in the test section separated by the end plates, is 0.9% (M1), 2.3% (M2) or 4.5% (M3). Overall, the experimental setup in the open test section in front of the outlet nozzle, including femur model, force balance mounting, and endplates has a blockage of 10.6% in total. The femur profiles have, due to their relatively high thickness, no supplement effect on the projected frontal area for angles up to 20°.

For the ground effect measurements, the blockage was constant at 16%, because the ground plate with the parallel translation was additionally positioned in the test section. In open test sections, blockage corrections are usually small (Barlow et al. 1999) and are commonly ignored (Cooper 1998), but a correction of the velocity can increase the significance of the measurements (Sayers and Ball 1983). Therefore, velocity measurements were taken at the femur profile with all experimental setup mounted, so that no further blockage correction must be applied.

### Lift and drag measurements

Lift and drag were not only measured for the AOAs observed for the femora of *Ecdyonurs* sp. but for a wider range of AOAs to allow comparison with the polars of common airfoils. The range of AOAs was − 40° to + 40°, with a step size of 2°. Positioning, measurement, and mean value calculation were automated by a customized Labview script (National Instruments, Austin, Texas). Each experimental case was repeated 10 times (*n* = 10). Lift and drag coefficients (*C*_L,_ *C*_D_) were calculated from the measured forces for lift (L) and drag (D) applying the standard formula for airfoils.

2 $${C}_{\rm{L}}=\frac{2 L}{ \rho S {U}^{2}}$$

3 $${{C}_{\rm{D}}= \frac{2 D}{ \rho S {U}^{2}}},$$

where *ρ* is the fluid density, *U* is the fluid velocity, and *S* is the profile surface area. *S* was calculated as the projected surface area. Forces were measured using a friction-free two-component balance with two independent platforms based on air-cushioned sledges and inductive displacement transducers (TR10, HBM GmbH, Darmstadt, Germany) at a sampling rate of 100 Hz at 2.5 m/s and 600 Hz at 6.7 m/s, respectively. The signal was recorded for 30 s and 5 s, respectively, by a A/D transducer (Spider 8, HBM, Darmstadt, Germany), low-pass filtered, and averaged at each data point.

### Ground effects

The thickness of the boundary layer ($\delta$) was estimated applying the Blasius equation for the case of laminar flow over a flat plate in a parallel direction to the free flow

4 $${{\updelta }=5\sqrt{\frac{ {\upnu } \text{x} }{U}}}, $$

where *ν* is the kinematic viscosity, *x* is the distance from the frontal edge of the plate, and *U* is the flow velocity. To estimate the boundary layer for *Ecdyonurus* sp. under natural conditions, we calculated the thickness for an assumed minimum case (*x* = 0.01 m, *U* = 2 m/s, water at 10 °C) and maximum case (*x* = 0.1 m, *U* = 0.1 m/s) by estimating the environmental extreme conditions. The resulting thickness of the boundary layer under natural conditions is 0.4–5.1 mm. In the videos, we observed the femora of living *Ecdyonurus* sp. at 0–2 mm above the ground, so that interactions with the boundary layer of the ground are probable. The ground clearance (*h*/*c*) can be calculated from the distance to the ground (*h*) and the chord length (*c*) (Fig. 2). The *h*/*c* was 0–2 under natural conditions.

A unilaterally tapered and strengthened ground board (PVC rigid foam, 250 × 200 × 3 mm, Fig. 2) was used to simulate the ground in the wind tunnel. The leading edge of the femur profile was positioned 100 mm behind that of the ground board. In accordance with Ahmed and Sharma (2005), we expected a maximal influence of the ground at *h*/*c* < 1. Femur model M1 was measured at Re = 1700 for a distance to the ground (*h*) of 4–60 mm (equates *h*/*c* 0.4–6.0). The boundary layer thickness estimated by Eq. (4) (*x* = 100 mm, *U* = 2.5 m/s, dry air at 20 °C) was 3.8 mm at the leading edge of femur model M1. Two AOAs (− 10°, − 20°) were tested, to include the range of possible AOAs (*n* = 10 for each angle and distance).

## Results

### The femur profile and angle of attack

Figure 1b shows a typical contour of the middle part of the femur. The shape of the cross section is not symmetrical. The upper half of the contour is relatively flat and shorter (89%) than the lower half, which is arched. On the upper half are indentations on each side. A profile of this shape can be expected to facilitate negative lift due to Bernoulli’s principle (Vogel 1994; Böswirth 2010).

The chord length (*c*) of the femur was 1.19 ± 0.13 mm (*n* = 10) and the length (*l*) was 2.80 ± 0.20 mm (*n* = 10). The maximum thickness of the profiles was located at 40–50% of the chord length. The morphologically possible maximal AOA of the *Ecdyonurus* femur measured − 31.3° ± 8.1° (*n* = 10) on average. The AOA had a negative value because the femur was tilted counterclockwise. In the other direction, the femur could be artificially tilted until it reached a positive AOA, which however is not a natural position.

In addition, we estimated the AOA from the images of living larvae taken in the flow tank. This video footage shows for the first leg pair AOAs from − 7° to − 15°. These data should be handled as estimates as it causes some errors to estimate AOA from 2D images. However, there is no doubt that *Ecdyonurus* sp. larvae are holding their femora at a negative AOA (Fig. 1a). At increasing flow speed, the larvae typically oriented themselves facing the current and position their first femora approximately perpendicular to the current (Supplement 2). The first femora were positioned 0–2 mm above the ground, with the first leg pair very close to the ground and increased distance for the femora of the 2nd to 3rd leg pair (Fig. 1a). The further calculations are based on this posture.

### Lift and drag

**Fig. 3:**
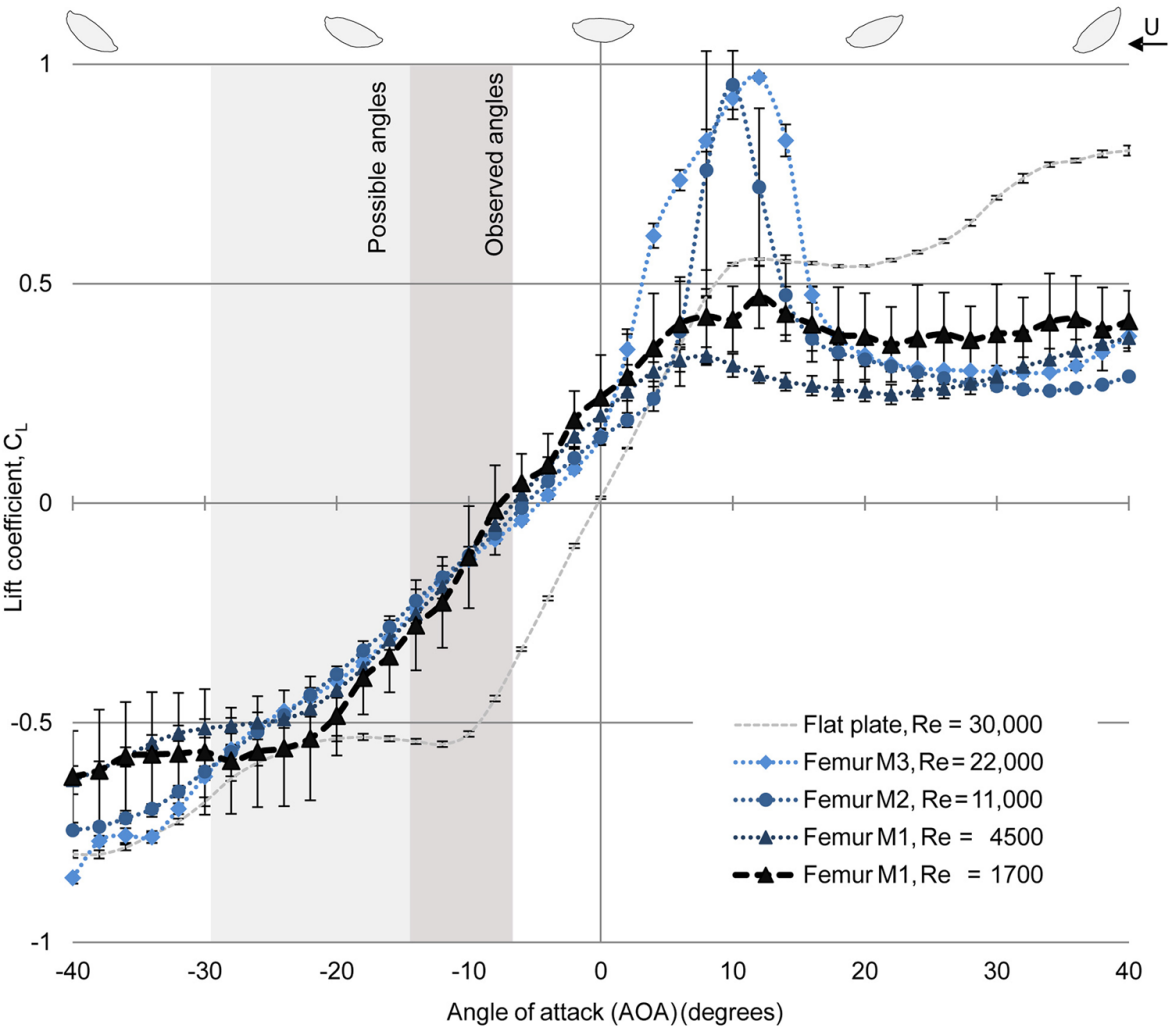
Lift coefficients (*C*_L_) of all femur models of *Ecdyonurus* sp. at different Reynolds numbers (Re) and angles of attack (AOAs). All values are means with standard errors (*n* = 10). At all Re, *C*_L_ increased linearly (0.03 *C*_L_/AOA (°)) between AOAs of − 26° and 0°. The standard errors of *C*_L_ were very small at Re ≥ 4500 but considerable at Re 1700

The *AOA* of the femur models had a strong impact on the lift (Fig. 3). For the biologically relevant condition (Re 1700), we found *C*_L_ from − 0.6 to 0.45 for the investigated range of AOAs (− 40° to 40°) with an almost linear increase at AOAs − 26° to 8°. For the range of AOAs determined from videos of living larvae (− 7° to − 15°), this correlated to *C*_L_ of − 0.3 to 0.05. The range of morphologically possible AOAs (up to − 30°) encompassed *C*_L_ till − 0.55.

The *C*_L_ showed approximately the same curve for AOAs between − 26° and 0° at all Re (1700–22,000), indicating that in this range of AOAs, the hydrodynamic effects are almost independent of the Re. At AOAs of − 26°, *C*_L_ was around − 0.5/− 0.55. Above an AOA of − 26°, the *C*_L_ increased almost linearly for all femur models and Re, reaching into positive lift (0.15–0.2) at AOAs of − 4° to − 8°.

Outside the range of AOAs of − 26° to 0°, the Re had a stronger impact on the *C*_L_ of the femur models. Below the AOA of − 26°, the *C*_L_ started to become considerably lower at higher Re (11,000, 22,000) in comparison to the lower Re (1700, 4500). For positive AOAs, *C*_L_ increased rapidly up to 1 at AOA around 10°, followed by a spontaneous collapse of lift (stall) at higher Re (11,000, 22,000). This effect was not observed at lower Re (4500, 1700) or negative AOAs. In comparison with the flat plate, the femur profile had a less extreme performance, which is reflected by the less steep slope of the *C*_L_ in the linear region of the curve.

**Fig. 4:**
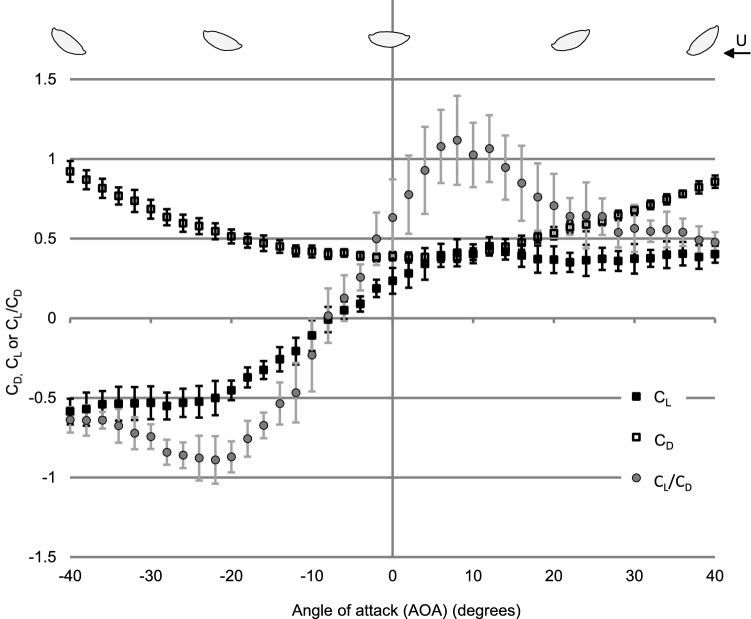
Drag coefficient (*C*_D_), lift coefficient (*C*_L_), and *C*_L_*/C*_D_ coefficients versus AOA for the femur model of *Ecdyonorus* sp. M1 at a Reynolds number of 1700. Values are means with standard errors (*n* = 10)

Aside from lift, we also measured the drag of the femur model for the biologically relevant condition (M1, Re 1700) (Fig. 4). *C*_D_ ranged from 0.92 to 0.38 for the tested range of AOAs. Drag was the lowest around an AOA of 2° and increased in both directions with increasing tilt (*C*_D_ 0.92 at AOA − 40°, *C*_D_ 0.86 at AOA + 40°). While there was a clear increase of drag with an increasing tilt, the increase of the *C*_D_ was very moderate between the AOAs of − 12° and + 10° (*C*_D_ 0–0.42) and still moderate at about AOA − 25° (*C*_D_ up to 0.55). In contrast, the *C*_L_ was strongly changing in the same range of AOAs. The ratio of lift to drag (*C*_L_*/C*_D_) allows the relative comparison of the development of both coefficients *C*_L_/*C*_D_. As we observe negative lift at negative AOAs, more negative *C*_L_*/C*_D_ describes a better hydrodynamic efficiency for the downward directed lift in comparison to drag. The stronger change in lift compared to the drag of the femur profile is clearly reflected in the steep slope of the *C*_L_*/C*_D_ ratio between AOAs of − 8° and − 22°. The magnitude of the *C*_L_*/C*_D_ ratio increases from 0.02 at AOAs of − 8° to − 0.89 at AOA of − 22°.

Moreover, the *C*_L_ reached higher magnitudes (up to − 0.6) for negative AOAs in comparison to positive AOAs (0.45). In consequence, the femora are well adapted to generate negative lift.

### Ground effects

**Fig. 5:**
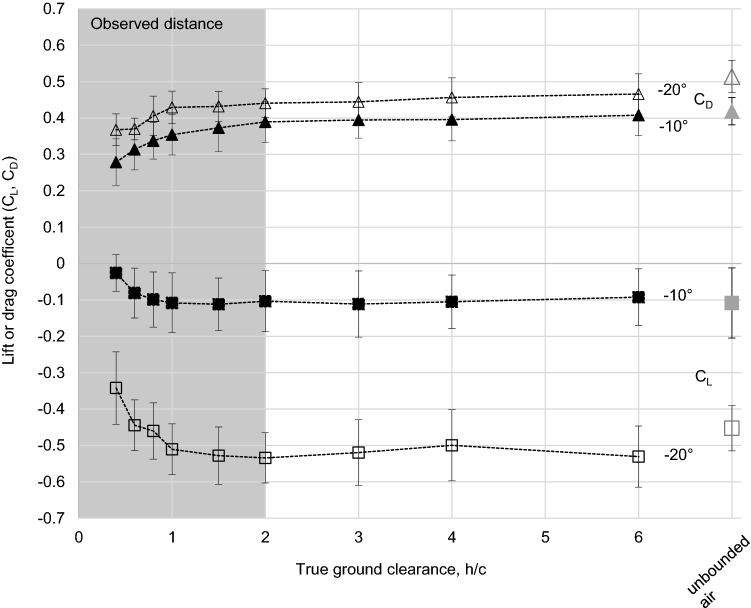
The lift (*C*_L_) and drag (*C*_D_) coefficient of the femur model (M1, Re = 1700) with increasing true ground clearance *h*/*c* (black symbols) and in unbounded air (grey symbols) for different angles of attack (AOA: − 10° and − 20°). All values are means with standard error (*n* = 10). The ground effect is visible for true ground clearance of *h*/*c* ≤ 2.0 showing decreasing *C*_L_ and *C*_D_ for lower *h*/*c*

The ground effect was investigated only for the biologically relevant case (Re 1700). The distance of the femur model to the ground had a clear impact on *C*_L_ and *C*_D_ (Fig. 5). For both coefficients, the investigated AOAs (− 10°, − 20°) (negative) lift and drag were considerably reduced in the direct proximity to the ground and increased consistently with the distance to the ground (*h*) until a true ground clearance (*h*/*c*) of around 2 was reached. At *h*/*c* ≥ 2, *C*_L_ and *C*_D_ seemed not to be impacted by the ground anymore and stayed almost constant. For all cases of ground clearance, the *C*_L_ was much more strongly affected by changes in the AOA than the *C*_D_.

**Fig. 6:**
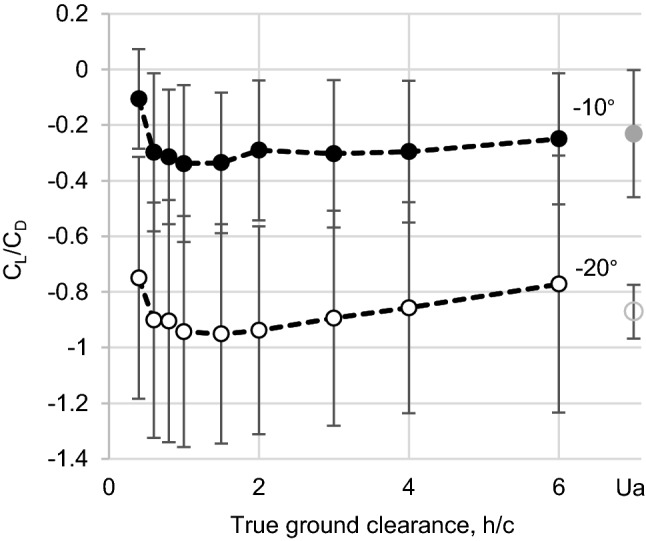
The lift-to-drag ratio (*C*_L_*/C*_D_) of the femur model (M1, Re = 1700, *n* = 10) with an increasing true ground clearance *h*/*c* (black symbols) and in unbounded air (grey symbols) for different angles of attack (AOA: − 10° and − 20°). A more negative *C*_L_/*C*_D_ ratio means a higher fluid-induced force in the direction to the ground. Most extreme values were attained at *h*/*c* 1–1.5 for both AOAs.

The *C*_L_*/C*_D_ ratio indicates better efficiency for the generation of (negative) lift at − 20° compared to − 10° AOA for all investigated distances to the ground and followed with this the same trend as in unbounded air (Fig. 6). The *C*_L_*/C*_D_ ratio is impacted by the ground clearance though. More negative *C*_L_*/C*_D_ values are observed with increasing *h*/*c* for both AOAs (− 10°, − 20°) until most negative values are reached at *h*/*c* 1.0–1.5. With further increasing *h*/*c, *the *C*_L_/*C*_D_ ratio slightly declines again. The most extreme values of the *C*_L_/*C*_D_ ratio were − 0.95 at AOA − 20° and − 0.35 at AOA − 10°.

## Discussion

The challenging flow environment, as well as the small size and sensitivity of the stream insects, often hinders direct measurements of the effects of current on stream insects. This is likely one of the reasons, why till today little is known about their morphological adaptations to current, despite the latter being the dominating factor of their environment. To overcome some of these challenges, we built a physical model of the femur and measured its lift and drag. While using a model always implicates simplifications in comparison to the natural conditions (e.g. occasional moving, slight changes in body orientation, eventual gill beating), the chosen approach gave us the possibility to control and change the AOA of the femur model and, thereby, gain a more comprehensive understanding of the basic acting principles in the biological system.

Considering the questions of this study, our results clearly show that the *Ecdyonurus* femora generate negative lift in the biologically relevant range of AOAs (at both, the biologically relevant Re and higher Re). The obtained *C*_L_ implicates more negative lift with an increasing tilt between AOAs − 26° to 0°. In combination with the femur’s morphologically possible range of AOAs, the results suggest that *Ecdyonurus* larvae can actively adjust the lift forces acting on them. *Ecdyonurus* larvae can be expected to experience positive lift forces caused by their dorso-ventrally flattened body shape. The negative lift generated by the femora can contribute to counterbalance positive lift caused by the overall body shape. This adjustment of the overall lift should be crucial for the larvae to stabilize their position in the flow, in particular in swifter currents, where lift forces increase considerably. This ability of the larvae to affect the experienced lift can explain the huge variation of total lift observed in previous studies on living *Ecdyonurus* larvae (Weissenberger et al. 1991). While changes in the overall body orientation of the living larvae could have impacted the measured overall flow forces too, they hardly can explain the negative lift. Moreover, the larvae are known to arrange themselves at higher flow speeds in a typical “flow facing” position, so that their overall body position should be approximately comparable. In the case of *Ecdyonurus* sp., the conspicuous head shield, which also has a negative AOA (Weissenberger et al. 1991), might produce additional negative lift forces contributing to keeping these mayfly larvae on the ground at higher flow speeds. The AOA of the headshield cannot be controlled by the larvae though, and, therefore, cannot explain the huge variation in the overall lift.

In fast flowing water, the femur’s cross-sectional shape can be compared with that of an airplane wing. The shape of the wing is known to affect the flow forces considerably. For the femur profile, between an AOAs of 0° and − 26, the magnitude of the (negative) lift increases linearly with the magnitude for the (negative) AOA at all investigated Re. At AOAs outside of this range, more extreme values can be recognized at higher Re in comparison to lower Re. For positive AOAs, lift increases rapidly for higher Re in our experiment (11,000, 24,000) followed by a sudden drop in lift (stall) at around AOA of 10°. In comparison, at the lower Re, lift shows only a very moderate stall. The same trend regarding stall was observed for NACA 0012 wing profile at a Re and AOA in about the same range (Laitone 1997; Alam et al. 2010). For the biologically relevant case, at negative lift, no stall was observed for the femur profile, which is a big advantage for the stability of the larvae. At relatively low Re, flat profiles have shown to have the highest *L*/*D* ratio (Laitone 1997), a fact which is reflected in our results in the steeper slope of the *C*_L_–AOA curve of the measured flat plate in comparison to the femur model (M3) (Fig. 3). However, in the case of the *Ecdyonurs* femur, a very flat profile would probably conflict with other morphological demands, such as the required space for the strong leg muscles required for locomotion in the current. Moreover, a highly sensitive reaction of lift to the AOA of the femur might not be favorable for the larvae, as it could cause problems while changing the body position due to common activities, such as feeding or locomotion. The shape of the femora of *Ecdyonurus* sp. is characterized by a convex bottom and an almost flattened upper side and seems to be adapted to use these effects to generate increased negative lift while also supporting other functions important for the larvae. Changing the AOA of their femora, and with this the amount of the experienced lift, enables the larvae to adjust to the changing flow conditions.

The generated negative lift comes with the cost of an increase in drag. However, the femur profile shows better hydrodynamic efficiency for the downward directed lift in comparison to drag, because the *L*/*D* ratio shows that the magnitude of lift increases proportionally more than drag with an increasing magnitude of the AOA for most of the biologically relevant range (AOA: − 30° to − 7°). It is likely much easier for the larvae to deal with drag than with lift. Ephemeroptera larvae have single claws helping them to attach to the substrates of the stream bottom (Ditsche-Kuru et al. 2012). In contrast to double claws, which can take advantage of clamp-like mechanisms, single claws are presumably less robust against detachment forces acting from varying directions. Single claws require a backward directed force to interlock with the substrate’s surface irregularities or biofilm (Ditsche-Kuru et al. 2012; Ditsche et al. 2014). Therefore, the generated drag might to some extent be useful for the larvae. The typical positioning of the tarsus, tibia, and femur (Fig. 1a) should result in a direct transfer of drag forces to the claws. This might also explain why the femora of the second and third leg pairs are usually arranged in a subsequently higher position. In this position, the following leg is not “protected” by the previous one, but is more exposed to the current, which results in the generation of both, more negative lift and more drag. While single claws work well in the direction of drag, they can be expected to have difficulties withstanding upwards directed lift forces. The latter could easily detach the animal from its support and cause its drift out of the habitat. In stronger currents, where the larvae orientate themselves in a position facing the flow, the femora of the first leg pair are usually positioned at the right angle to the middle axis of the larva, while the femora of the second and the third leg pair show increasingly larger angles to the middle axis (cf. Supplement 2). This positioning should make the larvae more resistant against forces acting to detach them from other than the main flow direction. This is important because, under natural stream conditions, the flow is usually not laminar, but rather turbulent. This means that water flow acts not only from the main flow direction but to some extent also from other directions.

**Fig. 7:**
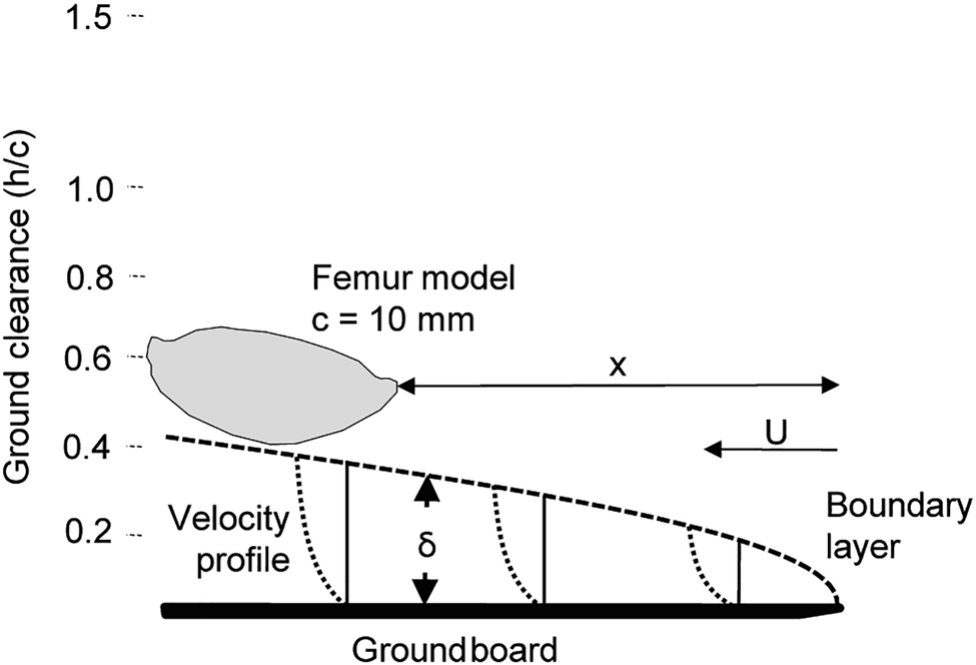
Schematic view of the boundary layer thickness (*ẟ*) and position of the femur model (*c* = 100 mm) at different ground clearances (*h*/*c*) during wind tunnel tests at Re = 1700. The calculated boundary layer thickness was 3.8 mm (equals *h/c* 0.38) at the position (*x*) of the femur model. Interaction of the model and boundary layer would only take place at *h*/*c* < 0.38. The thickness of a laminar boundary layer on a flat plate and several velocity profiles within it are estimated according to Vogel (1994)

Stream-inhabiting mayflies are benthic and their proximity to the substrate affects the flow forces that these larvae experience. While different fluid dynamic aspects can play a role in the proximity to the ground, the wind tunnel measurements of the femora profiles reflect the sum of all forces. In the direct proximity to the ground, both *C*_L_ and *C*_D_ were reduced, while their values increased with increasing ground clearance till *h*/*c* 1.5–2. A possible explanation for this could have been that the boundary layer with its reduced flow velocity causes the observed effect. However, calculating the thickness of the boundary layer using Eq. (4) (3.8 mm, equaling *h/c* 0.38 shows that all our measurements in the wind tunnel (ground distance 4–60 mm) were outside the boundary layer. Consequently, the lower *C*_L_ and *C*_D_ values up to *h/c* < 1.5–2 cannot be explained by the influence of the boundary layer (Fig. 7).

The decrease of *C*_L_ and *C*_D_ with an increasing proximity to the ground was in contrast to our expectations suggesting a downwards directed force for the femur profile due to Bernoulli effects. The wing-in ground effect is usually considered to reduce drag and to increase (positive) lift, for wing shapes, which are convex at the bottom side, but at very low or negative AOAs the increasing lift points in a downwards direction (negative lift) (Abramowski 2007). In the latter case, a Venturi nozzle is generated between the foil and the ground, so the negative lift is due to the Bernoulli effect. This effect is used by race cars and is caused by the Venturi nozzle between airfoil and ground, where high-speed low-pressure air sucks the airfoil down. For car undertrays, it was calculated (Jones and Smith 2003) that distances from the ground larger than the thickness of the boundary layer have negligible effects on lift and drag. Maybe the latter (profiles larger than the boundary layer) is the reason why no increased negative lift was observed. Moreover, the tilted position of the femur model changes the profile shape which approaches the bottom, which could change the circulation around the profile. Further measurements, such as flow visualization will be needed to better understand the observed effect in our study. However, the first femur was most of the time over an *h/c* of 1, and the second and third femora were always *h/c* > 2, and therefore lift was just slightly or not affected by the proximity to the ground.

Other torrenticol insects can be expected to take advantage of the same mechanism to generate negative lift with their legs supporting them to stay on the ground. This seems especially likely for several stream mayfly larvae as different taxa with dorso-ventrally flattened body shape and widened femora can be found in running waters all over the world. These stream mayfly species belong not only to the heptageniid family (e.g. *Epeorus assimils*, *Iron alpicola*, Europe; *Rhitrogena* sp., Europe and North America), but also to other families, such as *Lepeorus* sp. (Leptophlebiidae, New Caledonia), *Kirrara* sp. (Leptophlebiidae, Australia) *Delatidium* sp. (Leptophlebiidae, New Zealand), *Penaphlebia* sp. (Leptophlebiidae, South America), or *Drunella* sp. (Ephemerellidae, North America). Several of these species developed strikingly similar body shapes (Hynes 1970; Peter et al. 1979; Campbell 1990).

Morphological adaptations of stream insects to the hydrodynamic forces can be very complex and reach far beyond simple adaptations of the overall body shape. Blackfly larvae can position most of their body in the lower boundary layer while their filtering fans are arranged in a way that they take advantage of the higher flow speed in the top of the boundary layer and initiated vortex formation, respectively (Chance and Craig 1986; Merritt et al. 1996). Water pennies use a specialized mechanism to delay the boundary layer separation and thereby reduce drag (Smith and Dartnall 1980). Aside from being a further example of elaborate flow adaptations, this study emphasizes the importance of considering all relevant flow forces (drag, lift, and in certain cases acceleration reaction force) (Denny et al. 1985; Ditsche and Summers 2014) to understand the interaction of stream insects with their flow environment. From *Ecdyonurus* larvae, one can learn that insect legs of a specific shape can be used like wings or fins to affect an animal’s position in a fluid-shaped environment. These legs function like hydrofoils helping the larvae to stay on the ground. The fluid dynamic characteristics of our 2D-wing model clearly show that the legs can generate negative lift and therefore act like hydrofoils. Applying the average measures of the first femora at Re = 1700, which is close to natural conditions (Re up to 1500), and an assumed AOA of − 15° lift and drag generated by the legs can be estimated using rearranged Eqs. (2) and (3). With an estimated lift of − 0.172 mN per leg for a flow velocity of 0.6 m/s, this results in a total of − 1 mN for all legs of the larva. While this is a rough estimate as it builds up on the assumptions of a specific Re, a specific AOA and a rectangular angle of the femur to the medial axes (what is not necessarily the case for the second and third femur), this is in the same range as the most negative lift measured on living larvae (− 1.2 mN) (Weissenberger et al. 1991). An increase of the flow velocity to 1 m/s would result in − 2.8 mN lift in total for all legs of the larva. Drag at 0.6 m/s for the same AOA would result in about 0.56 mN for all 6 legs. This compares well to 1.6 mN calculated for the same flow speed on the basis of the *C*_D_ given for the whole *Ecdyonurus* larvae by Weissenberger et al. (1991) considering that the other body parts are causing drag, too. A heptageniid larval claw can easily withstand drag force of 0.093 mN per leg on even slightly rough substrates and much higher forces on rougher surfaces (data from *Epeorus assimilis*) (Ditsche et al. 2014). Aside from spotting light on the complexity of morphological adaptations in stream insects to flow, our results suggest that such elaborated adaptations could be an interesting model for bio-inspired design. We might be able to learn some features for further technical applications, for example to improve attachment in water current exposed places.

## Supplementary Information

Below is the link to the electronic supplementary material.

Supplementary file1 (JPG 77 kb)

Supplementary file2 (MP4 7851 kb)

## Data Availability

The data are available from the first author upon request.
