# Supplementary figures and images for: Hydrofoil-like legs help stream mayfly larvae to stay on the ground
Source: J Comp Physiol A Neuroethol Sens Neural Behav Physiol. 2023 Feb 26;209(2):325–36. doi: 10.1007/s00359-023-01620-2 (PMC10006037; doi:10.1007/s00359-023-01620-2)

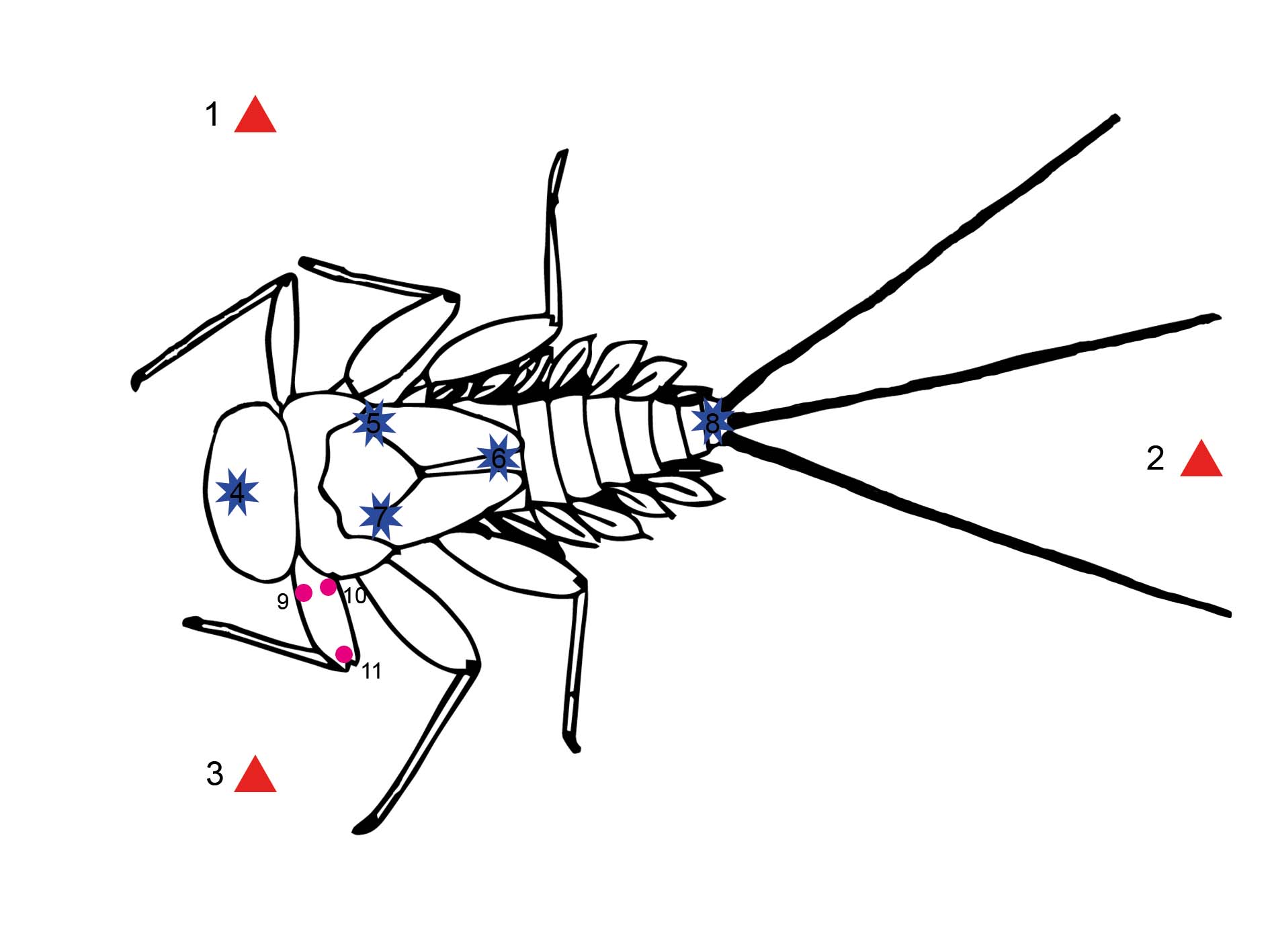

Supplement: Supplementary file 1 — Supplementary file1 (JPG 77 kb) [file 359_2023_1620_MOESM1_ESM.jpg]
